# MRI-visible perivascular spaces as an imaging biomarker in Fabry disease

**DOI:** 10.1007/s00415-020-10209-7

**Published:** 2020-10-19

**Authors:** D. Lyndon, I. Davagnanam, D. Wilson, F. Jichi, A. Merwick, F. Bolsover, H. R. Jager, L. Cipolotti, C. Wheeler-Kingshott, D. Hughes, E. Murphy, R. Lachmann, D. J. Werring

**Affiliations:** 1 Stroke Research Centre, Department of Brain Repair and Rehabilitation, Institute of Neurology, Russell Square House, London, UK; 2 Department of Brain Repair and Rehabilitation, UCL Institute of Neurology, Queen Square, London, UK; 3 Department of Biostatistics, University College of London, London, UK; 4 Charles Dent Metabolic Unit, National Hospital for Neurology and Neurosurgery, London, UK; 5 Department of Neuropsychology, National Hospital for Neurology and Neurosurgery, Queen Square, London, UK; 6 Department of Neuroinflammation Queen Square MS Centre, UCL Institute of Neurology, London, UK; 7 Lysosomal Storage Disorders Unit, Royal Free Hospital, Rowland Hill Street, London, UK; 8 New Zealand Brain Research Institute, Christchurch, New Zealand

**Keywords:** Fabry disease, Magnetic resonance imaging, Perivascular spaces, Neurovascular disease

## Abstract

**Introduction:**

Fabry disease (FD) is an X-linked lysosomal storage disorder resulting in vascular glycosphingolipid accumulation and increased stroke risk. MRI findings associated with FD include white matter hyperintensities (WMH) and cerebral microbleeds (CMBs), suggesting the presence of cerebral small vessel disease. MRI-visible perivascular spaces (PVS) are another promising marker of small vessel disease associated with impaired interstitial fluid drainage. We investigated the association of PVS severity and anatomical distribution with FD.

**Patients and methods:**

We compared patients with genetically proven FD to healthy controls. PVS, WMH, lacunes and CMBs were rated on standardised sequences using validated criteria and scales, blinded to diagnosis. A trained observer (using a validated rating scale), quantified the total severity of PVS. We used logistic regression to investigate the association of severe PVS with FD.

**Results:**

We included 33 FD patients (median age 44, 44.1% male) and 20 healthy controls (median age 33.5, 50% male). Adjusting for age and sex, FD was associated with more severe basal ganglia PVS (odds ratio (OR) 5.80, 95% CI 1.03–32.7) and higher total PVS score (OR 4.03, 95% CI 1.36–11.89). Compared with controls, participants with FD had: higher WMH volume (median 495.03 mm^3^ vs 0, *p* = 0.0008), more CMBs (21.21% vs none, *p* = 0.04), and a higher prevalence of lacunes (21.21% vs. 5%, *p* = 0.23).

**Conclusions:**

PVS scores are more severe in FD than control subjects. Our findings have potential relevance for FD diagnosis and suggest that impaired interstitial fluid drainage might be a mechanism of white matter injury in FD.

## Introduction

Fabry disease (FD) is an X-linked lysosomal storage disorder caused by a deficiency in the α-galactosidase A (GLA) enzyme, leading to the accumulation of globotriaosylceramide (Gb3) in multiple organ tissues and blood vessels, with increased stroke risk [1]. Ischaemic stroke and transient ischaemic attack are the most prevalent central nervous system manifestations of FD [2]. The pathophysiology of cerebrovascular dysfunction in FD is complex, involving effects on blood flow, vessel walls, and blood components. Cerebral small-vessel disease (SVD) is common, causing clinical subcortical ischaemic stroke or asymptomatic brain imaging changes, specifically white matter hyperintensities (WMH), lacunes and cerebral microbleeds (CMBs) [3].

Arterial remodelling also leads to medium and large vessel disease including dolichoectasia [3]. WMH are the commonest brain imaging marker in FD, but are not specific and can be present in other conditions, for example multiple sclerosis, leading to misdiagnosis [4]. Laboratory testing for FD is guided by clinical and radiological suspicion, including the presence of SVD markers; in males, FD can be diagnosed by measuring alpha-galactosidase A activity, while in females, only the identification of a pathogenic mutation in the GLA gene allows a definite diagnosis.

MRI-visible perivascular spaces (PVS) are another promising marker of SVD that might be relevant for diagnosing and understanding mechanism of white matter injury in FD. One plausible underlying mechanism of enlarged PVS is impaired fluid drainage caused by protein and other debris; in lysosomal storage disorders such as the mucopolysaccharidoses (MPS), widened PVS containing a meshwork of fibrotic tissue and glycolipid filled neurons have been described [5]. We therefore hypothesised that PVS scores will be higher in FD patients compared to a control population.

## Methods

### Participant recruitment

Patients with a genetically confirmed diagnosis of Fabry disease aged 18 or over attending the Charles Dent Metabolic Unit at the National Hospital for Neurology and Neurosurgery or the Lysosomal Storage Disorders Unit at the Royal Free Hospital (*n* = 30) were screened for study eligibility between February 2012 and July 2013.

Participants were excluded if they met any of the following criteria: i) age < 18 years, ii) another central nervous system (CNS) disease (e.g. epilepsy or multiple sclerosis; *n* = 5) or intellectual disability (*n* = 1) in addition to Fabry disease, iii) ineligible to have a 3 T MRI scan (mainly due to cardiac devices or renal devices/transplant; *n* = 22).

Eligible patients were recruited by self-selected sampling through contact by telephone or whilst attending clinic. They were provided with a study information sheet and invited to contact the researcher if they would like to participate. Healthy volunteer control participants aged 18 or over were recruited by advertisement by poster between August 2012 and July 2013.

### Standard protocol approvals, registrations and patient consent

We obtained Informed written consent from all participants and the protocol was reviewed and approved by the NHS London Bromley Research Ethics Committee and the NHS UCLH/UCL Joint Biomedical Research Unit.

### Study setting and patient population

Patients over 18 years old with genetically confirmed FD attending the Charles Dent Metabolic Unit at the National Hospital for Neurology and Neurosurgery or the Lysosomal Storage Disorders Unit at the Royal Free Hospital between February 2012 and July 2013 were recruited for the study. Healthy volunteers were recruited by poster between August 2012 and July 2013. FD patients were excluded if they had: (1) a diagnosis of another central nervous system disease; (2) intellectual disability; or (3) contraindications to undergoing MRI, for example an implanted cardiac device.

### Neuroimaging acquisition and analysis

We scanned all participants using a standardised brain MRI protocol performed on a 3 T Achieve TX MRI system (Philips Healthcare, Best, The Netherlands) using a Sensitivity Encoding (SENSE) 32-channel coil. The parameters of the sequences were as follows:

1. 3D Axial T1-weighted gradient turbo field echo (TFE) sequence, TFE factor 230, Shot interval: 3000 ms, reconstructed slice thickness: 1 mm isotropic with no slice gaps, 256 mm × 160 mm × 256 mm (AP × RL × FH) field of view (FOV);
2. axial T2-weighted dual echo fast field echo (FFE) sequence, first echo proton-density (PDT2) TE: 20.7 ms, second echo TE 85 ms, TR 6000 ms, slice thickness: 2 mm with no slice gaps, 240 mm × 180 mm × 144 mm (AP × RL × FH) FOV;
3. axial phase sensitive inversion recovery (PSIR) turbo spin echo (TSE) sequence, TE 13 ms, inversion recovery delay: 400 ms, slice thickness: 2 mm with no slice gaps, 240 mm × 180 mm × 144 mm (AP × RL × FH) FOV;
4. 3D axial susceptibility weighted imaging (SWI) sequence, TE: shortest (automatic), TR: shortest (automatic), flip angle: 10°, slice thickness: 1 mm with no slice gaps, 240 mm × 180 mm × 180 mm 144 mm (AP × RL × FH) FOV.

Readers trained in assessing MRI neuroimaging analysed the images systematically for the following potential biomarkers of FD criteria outlined by the STRIVE consensus paper, [6] blinded to clinical details: white matter hyperintensities (WMH), perivascular spaces (PVS), microbleeds and lacunes.

**Fig. 1:**
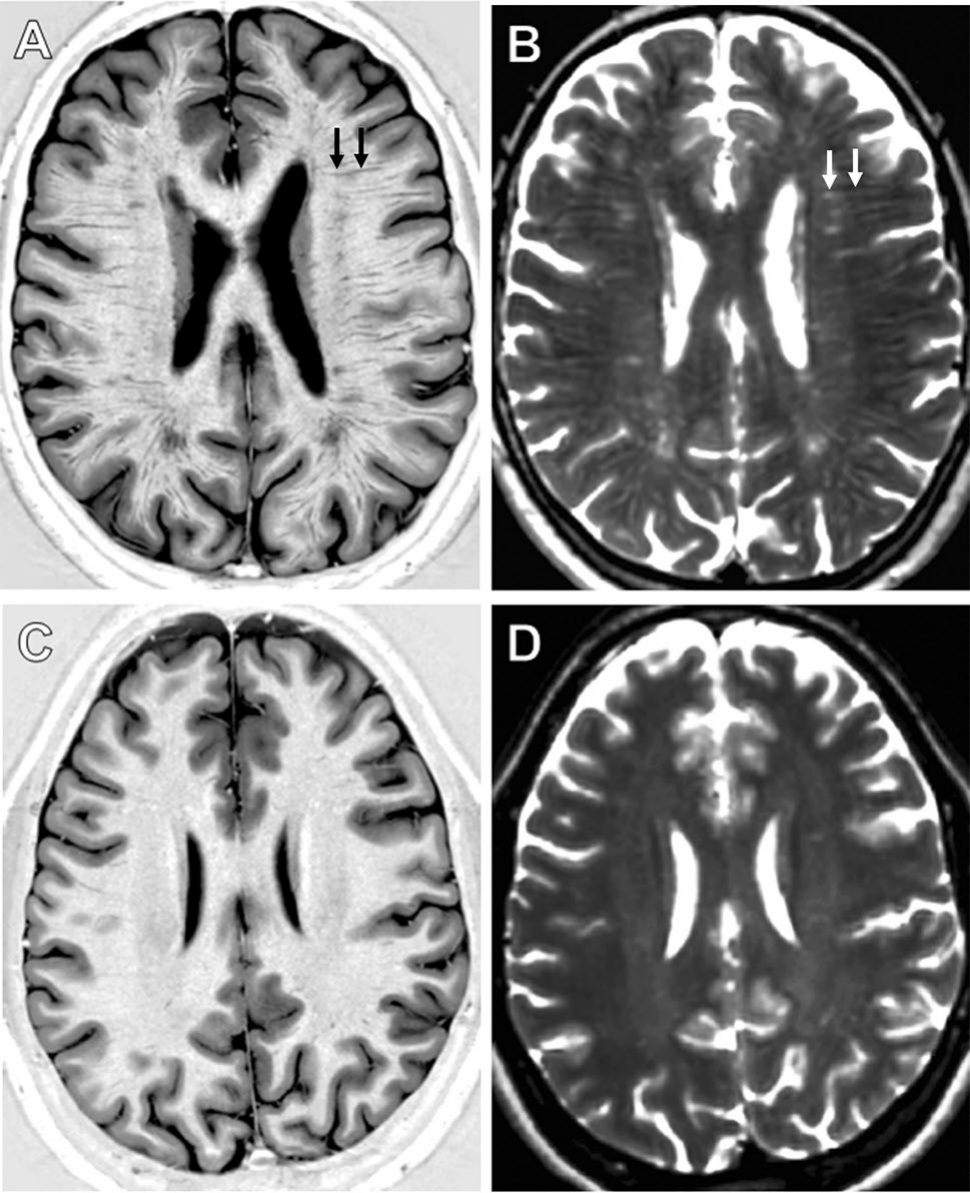
Examples of severe and non-severe perivascular space enlargement in our study population. **a** and **b** Phase-Sensitive Inverstion Recovery (PSIR) and T2-weighted axial images of a subject with a severe PVS score in our FD group. On the right panel (**a**) a PVS is shown by black arrows; on the left panel (**b**) a PVS is shown by white arrows. **c** and **d** PSIR and T2-weighted axial images of a subject with a mild PVS score in our control group

We rated perivascular spaces on axial T2 and PSIR sequences using a validated five-point visual rating scale (0 = no PVS, 1 ≤ 10 = 1, 11 ≤ 20 = 2, 21 ≤ 40 = 4) at the level of the basal ganglia (BG), and the centrum semiovale (CS) [7]. Midbrain PVS (MB-PVS) were rated on a two-point scale (0 = no PVS, 1 = PVS present). The slice with the highest number of enlarged PVS was selected after reviewing each anatomical area and an enlarged PVS score calculated. Figure 1 provides examples of severe and non-severe perivascular space enlargement in our study population on Phase-Sensitive Inversion Recovery (PSIR) and T2-weighted axial MRI sequences.

Microbleeds were rated on susceptibility weighted imaging (SWI) sequences using the Microbleed Anatomical Rating Scale (MARS). [8] Lacunes were rated on axial T2 and PSIR sequences using simple frequency counts. [6] White matter lesions were assessed on T2-weighted sequences and segmented.

A semi-automated method of region of interest (ROI) placement of WMH was carried out using JIM version 5.0 (Xinapse Systems, Northants) in which ROIs were assigned automatically and then adjusted by hand. Total ROI volumes were then automatically calculated and tabulated.

Fabry International Prognostic Index (FIPI) composite severity scores were calculated for each FD patient using clinical data collected from patient records including clinical presentation electrophysiology and blood results. [9]

### Statistical analysis

Statistical analysis was carried out using Stata 13.0 (StataCorp, USA). Data were first tested for normality using histogram analysis. Ordinal data were tested to ensure it did not violate the proportional odds assumption to allow ordinal logistic regression. Univariate analysis of PVS scores was carried out using binary logistic regression for binary outcomes (MB-PVS scores), and ordinal logistic regression for the BG-PVS, CS-PVS and total PVS scores. Multivariate analysis was then carried out using the same methods with age and sex included as covariates. White matter hyperintensity volume data were testing using Wilcoxon rank sum tests and Fisher exact tests were carried out on the scores collected for the other SVD markers as appropriate.

## Results

Thirty-three FD patients and 20 healthy controls were included in this study. The median age was 44 (interquartile range (IQR) 30–54) in the FD group and 33.5 (IQR 30.5–42) in the control group; there was a higher proportion of males in the control group (50% vs. 44%) (Table 1). Two patients in the FD group had a history of ischaemic stroke and another patient had a previous transient ischaemic attack (TIA) compared to none of the control group. For those with FD, the median Fabry International Prognostic Index (FIPI) composite severity score was 1 out of a total maximum possible score of 7; subdomain scores are listed in Table 2.

**Table 1:** Study population characteristics in FD and healthy control groups

	Fabry group *n* = 33	Control group *n* = 20
Age, median (IQR)	44.0 (30–54)	33.5 (30.5–42)
Sex: male, *n* (%)	15 (45.45%)	10 (50%)
Hypertension, *n* (%)	8 (23%)	2 (10%)
Diabetes, *n*	0	0
BG-PVS, median (IQR)	1 (1–2)	1 (1–1)
CS-PVS, median (IQR)	3 (2–3)	2 (2–3)
MB-PVS, median (IQR)	1 (1–1)	1 (0–1)
Total PVS, median (IQR)	5 (4–6)	4 (3–5)
WMH Volume (mm^3^), median (IQR)	495.03 (92.05–1480.55)	0.00 (0–199.98)
Lacune presence, *n* (%)	7 (21.21%)	1 (5%)
CMB presence, *n* (%)	7 (21.21%)	0

BG-PVS, CS-PVS, MB-PVS and Total PVS indicate basal ganglia, centrum semiovale, midbrain and total perivascular space scores respectively. *WMH* indicated white matter hyperintensity. *CMB* indicates cerebral microbleeds; *IQR* indicates interquartile range

**Table 2:** Distribution of disease severity in the Fabry disease group indicated by Fabry International Prognostic Index (FIPI) score and plasma globotriaosylceramide (Gb3) concentration

Domain	Score	Maximum possible score
Cardiac score, median (IQR)	1.5 (0–2.5)	6.5
Renal score, median (IQR)	2 (0–3.5)	7
Neurological score, median (IQR)	1.5 (0–3)	7
Risk of death, median (IQR)	2 (0–2)	6
Composite score, median (IQR)	1 (0–3)	7
Plasma Gb3 (µmol/L), median (IQR)	14.07 (4.34–32.23)	–

*IQR* indicates interquartile range, *SD* indicates standard deviation

### Descriptive data

The FD group had higher median CS-PVS scores (3 (IQR = 2–3) versus 2 (IQR 2–3)) and higher total PVS severity scores (5 (IQR 4–6) versus 4 (IQR 3–5)) (Table 1). Median BG-PVS and MB-PVS scores were similar in both groups.

**Fig. 2:**
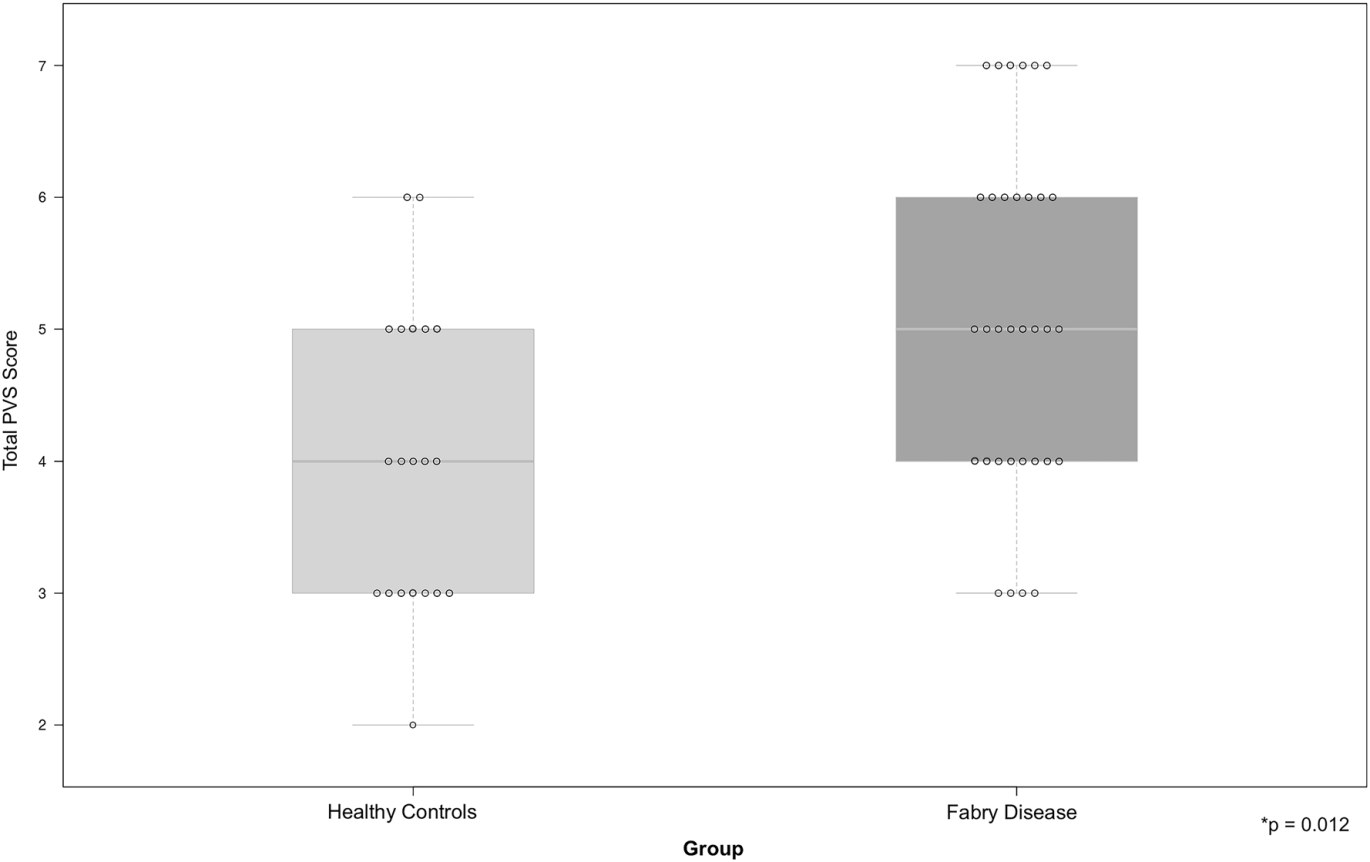
Dot and box plots showing total perivascular space scores in the Healthy control and Fabry disease groups. Total perivascular space scores were significantly higher in the Fabry disease group when compared with the Healthy control group (5 [IQR = 4—6] versus 4 [IQR 3—5], *p* = 0.012)

Total WMH volume was higher in the FD group (median 495.03 mm^3^ (IQR = 92.05–1480.55) versus 0 mm^3^ (IQR = 0–199.98), *p* = 0.0008 (Fig. 2).

Seven patients (21.21%) with FD had CMBs compared with 0 in the control group, *p* = 0.04. Seven patients in the FD group had lacunes (21.21%) compared with 1 participant in the control group (5%), *p* = 0.23.

### PVS statistical models

Our data did not violate the proportional odds assumption on likelihood ratio testing allowing us to proceed with ordinal regression analysis. In univariate models, patients with FD had a significantly increased odds of higher CS-PVS scores (OR 3.18; 95% CI 1.10–9.20), total brain PVS scores (OR 4.46; 95% CI 1.56–12.76) and BG-PVS scores (OR 7.5; 95% CI 1.49–37.66) compared with controls. FD was also associated with midbrain-PVS (OR 3.11; 95% CI 0.75–12.81).

In multivariate models correcting for age and sex (Table 3), FD was independently related to increased BG-PVS score (OR 5.80; 95% CI 1.03–32.7) and total PVS score (OR 4.03; 95% CI 1.36–11.89) but not CS-PVS score (OR 2.70; 95% CI 0.92–7.98) or MB-PVS score (OR 3.31; 95% CI 0.69–15.88) (Table 3).

**Table 3:** Multivariable models showing odds of increased PVS severity scores in patients with Fabry disease versus controls

SVD Marker	Model variable	OR	95% CI	*P* value
BG-PVS	Fabry vs control	5.80	1.03–32.70	0.046
	Age	1.07	1.01–1.13	0.008
	Sex	1.62	0.40–6.64	0.499
CS-PVS	Fabry vs control	2.70	0.92–7.98	0.072
	Age	1.04	1.00–1.08	0.076
	Sex	1.37	0.49–3.80	0.543
MB-PVS	Fabry vs control	3.31	0.69–15.88	0.134
	Age	1.01	0.95–1.08	0.654
	Sex	5.55	0.99–31.25	0.052
Total PVS	Fabry vs control	4.03	1.37–11.89	0.012
	Age	1.05	1.01–1.09	0.017
	Sex	2.21	0.81–6.03	0.123

BG-PVS, CS-PVS, MB-PVS and Total PVS indicate basal ganglia, centrum semiovale, midbrain and total perivascular space scores, respectively. *IQR* indicates interquartile range, *OR* indicates odds ratio

## Discussion

We found an independent association between FD and enlarged PVS, providing a potential new diagnostic biomarker with mechanistic relevance for understanding FD pathophysiology. Our findings are consistent with other studies showing associations of other SVD markers with FD, including WMH, lacunes and CMBs [3]. In adjusted models, total PVS scores and BG-PVS scores were increased in FD patients, but this was not statistically significant for CS-PVS and MB-PVS. We confirmed the findings of previous studies that CMBs and more severe WMH are associated with FD [10].

Enlarged PVS are emerging as a consistent imaging biomarker marker in SVD, with potential clinical relevance [7]. In sporadic SVD, BG-PVS have reported to correlate with cognitive dysfunction [11]. Recent data suggest that cognitive impairment is common in FD, so the functional relevance of PVS for cognition in FD is a topic for further investigation [12]. Enlarged PVS also seem to occur earlier in the natural history of SVD, before WMH burden is increased, [13] so might be an early clue to FD, potentially aiding differentiation from other radiological “mimics” (e.g. multiple sclerosis) in young people [14]. We believe, however, that the mechanism of both WMH and PVS are likely to be different in FD that conventional SVD. The median age of FD patients in our cohort was 44, much lower compared to the population of conventional SVD.

The periarterial drainage pathways in the walls of cerebral arteries are emerging as major routes of toxin and metabolic waste clearance from the brain that might be impaired in sporadic forms of SVD such as hypertensive and cerebral amyloid angiopathy (CAA) [15, 17]. Proposed mechanisms in CAA include amyloid-β deposition leading to retrograde perivascular space enlargement in the underlying white matter due to obstruction of normal perivascular fluid flow [18]. Other mechanisms suggested for enlarged PVS in SVD include: blood brain barrier dysfunction; ex-vacuo dilatation due to white matter atrophy; and loss of the normal ‘vascular pump mechanism’, whereby pulsation of the cerebral arterioles promoting flow along an adjacent PVS is reduced due to arteriolosclerotic stiffening [10].

Our findings suggest that enlarged PVS are a feature of FD. Indeed, other lysosomal storage diseases are also associated with PVS enlargement; in mucopolysaccharidosis (MPS), enlarged PVS in the basal ganglia are common [5]. Pathological studies in MPS have described enlarged PVS containing a meshwork of fibrotic tissue and storage filled cells as well as deposition of substrate within the leptomeninges, leading to obstructed CSF flow and retrograde PVS enlargement [20, 21]. Several post-mortem pathology studies have been reported in FD patients with glycosphingolipid found within the brain neurons, vessels and meninges but we found none that investigated glycoshingolipid deposition along PVS [22].

Our data do not provide definitive evidence for a mechanism of PVS enlargement in FD, however, the possible preferential involvement of the BG suggests a shared mechanism with that proposed in MPS. It has been widely found that hypertensive SVD also preferentially affects the small vessels of the basal ganglia and deep circulation; a similar susceptibility of these vessels as a cause for PVS enlargement in FD should be a target for future investigation [23].

There are some limitations of this study. With a cross-sectional study design, we have shown a clear association but cannot definitely prove that FD causes PVS enlargement. The patients in this study were recruited by consent through specialist units, therefore, may be subject to some selection bias. The patients in our FD group have relatively mild clinical disease as demonstrated by their Fabry International Prognostic Index scores (see Table 2), and with only two patients having a history of ischaemic stroke, one a previous ICH, and another a previous TIA. This may be partly because patients with implanted cardiac devices were excluded due to contraindications to MRI, which may have excluded patients with more severe systemic involvement. However, this bias would be expected to reduce the chance of us finding an association of FD with PVS. Finally, although the STRIVE paper defines lacunes and PVS on FLAIR imaging, our protocol did not include this. [5] Instead, we used T2-weighted and PSIR images, on which PVS can still be distinguished from lacunes (indeed, PSIR seems to be a promising sequence with which to identify PVS).

The difference in PVS scores between Fabry patients and controls was not large, which again may be a reflection of the mild severity of Fabry disease in our patient study group. PVS is a new biomarker in FD that plausibly relates to disease mechanisms. The independent added diagnostic value of PVS remains to be established in studies of FD compared to conditions with which it could be confused, for example, MS and that there may be added value as a biomarker in conjunction with the established WMH, microbleed and lacune biomarkers.

Finally, although our cohort is genetically proven and larger than some previous studies, the study population remains small and the large confidence intervals found in the statistical analysis suggest that our study cannot definitely confirm a predilection for enlargement of BG-PVS rather than CS-PVS in FD.

## Conclusion

To the best of our knowledge, ours is the first report describing enlarged PVS in FD. Furthermore, our findings are in a group of patients with relatively mild FD severity who could undergo MRI. Our findings could be explained by glycosphingolipid deposition in the PVS causing impaired white matter interstitial fluid drainage. Enlarged PVS are a promising new imaging marker with potential relevance in FD for diagnosis and understanding disease mechanisms.
